# Socioeconomic Inequalities in Poor Health-Related Quality of Life in Kermanshah, Western Iran: A Decomposition Analysis

**Published:** 2018-01-27

**Authors:** Satar Rezaei, Mohammad Hajizadeh, Masoud Khosravipour, Farid Khosravi, Shahab Rezaeian

**Affiliations:** ^1^Research Center for Environmental Determinants of Health, School of Health, Kermanshah University of Medical Sciences, Kermanshah, Iran; ^2^School of Health Administration, Faculty of Health, Dalhousie University, Halifax, Canada; ^3^Student Research Committee, Kermanshah University of Medical Sciences, Kermanshah, Iran

**Keywords:** Inequalities, Socioeconomic status, Health-related quality of life, Iran

## Abstract

**Background:** Socioeconomic status (SES) is an important determinant of health-related quality of life (HRQoL). We aimed to quantify socioeconomic-related inequality in poor-HRQoL among adults in Kermanshah, western Iran.

**Study design:** A cross-sectional study.

**Methods:** Overall, 1730 adults (18-65 yr) were selected using convenience sampling from Kermanshah, Iran. A self-administrated questionnaire was used to collect data on socio-demographic characteristics, SES, lifestyle factors and HRQoL of participants over the period between May and Aug 2017. The concentration curve and concentration index (C) were used to illustrate and measure wealth-related inequality in poor-HRQoL. Additionally, we decomposed the C index to identify factors explaining wealthrelated inequality in poor-HRQoL.

**Results:** The overall prevalence of poor-HRQoL was 35.3% (95% confidence interval[CI]: 33.1%, 37.6%). The poor-HRQoL was mainly concentrated among the poor adults (C=-0.256, 95% CI: -0.325, -0.187). Poor-HRQoL was concentrated among men (C=-0.256, 95% CI: -0.345, -0.177) and women (C=-0.261, 95% CI: -0.310, -0.204). Wealth, physical inactivity, the presence of chronic health condition(s), lack of health insurance coverage were the main factors contributing to the concentration of poor-HRQoL among socioeconomically disadvantaged adults.

**Conclusions:** Socioeconomic-related inequalities in poor-HRQoL among adult should warrant more attention. Policies should be designed to not only improve HRQoL among adults but also reduce the prorich distribution of HRQoL among adults in Kermanshah.

## Introduction

Inequalities in health among different social groups are a major public health problem ^1^. Inequalities in health exist for a wide range of health measures and for many indicators of the social position including, wealth, income and education^2^. Social inequalities in health can result from differences in healthy behaviors and/or inequalities in access to healthcare services across different groups. However, social determinants of health play an important role in explaining unequal distribution of poor health outcomes across the population ^1,3-5^.

Extent work has documented systematic differences in health by socioeconomic status (SES) in different countries^6^. A statistically significant relationship between SES (e.g., wealth, income, education, education level, and employment status) and health-related quality of life (HRQoL) has been observed in several countries ^7-12^. The unequal distribution of HRQoL across different social groups is an important policy objective. Measuring and identifying factors affecting the distribution of HRQoL is essential for the design and implementation of the effective policies to reduce social inequalities in health ^11,13^.

Income level, years of education, marital status, physical activity, smoking behavior, health insurance coverage and having chronic health condition(s) were identified as main determinants of HRQoL among adults in the western region of Iran^14^.

Although the main determinants of HRQoL across different social groups are well documented, to the best our knowledge, there is no study in Iran that measures socioeconomic-related inequalities in HRQoL in Iran. We used the concentration index (C) approach to quantify wealth-related inequalities in poor-HRQoL in Kermanshah city, western Iran. Further, we decomposed the C index to identify factors explaining wealth-related inequality in poor-HRQoL. The results of this study enable health-policymakers to design and implement interventions to reduce inequalities in health among Iranian adults.

## Methods

### Study setting

This study was carried out in Kermanshah city, the capital of Kermanshah Province, western Iran. This province consists of fourteen counties. It is bordered by Kurdistan Province to the north, Ilam Province to the south, Hamadan and Lorestan Provinces to the east and Iraq to the west. The total population of Kermanshah city was estimated to be approximately one million in 2016.

### Study population, sample size, and sampling method

This cross-sectional study was conducted from May to Aug 2017. We collected information on the socio-demographic, socioeconomic, behavioral risk factors and poor-HRQoL of adults (18-65 yr old) in Kermanshah city. The following formula was used to calculate the sample size:

(1) $$n=\;\frac{Z^{2}\;p\;\left(1-p\right)}{d^{2}}.$$

Where n shows the required samples size at the 95% level of significance, p indicates the prevalence of poor-HRQoL among the general population (set to a value of 0.5) and d is the degree of precision (set at 0.05). We added 30% to the required sample size of 1537 and collected information from 1983 adults. To select our participants, we first divided the Kermanshah city into five areas of central, western, eastern, southern and northern. Then, equal samples were drawn from each study area by using convenience sampling technique.

### Data collection and variables

A self-administrated questionnaire, developed and validated in a previous study^8^, was used for data collection. The questionnaire was divided into two parts. The first part included questions about age, gender, marital status, years of education, employment status, health insurance coverage, the presence of chronic health condition(s), physical activity, smoking behavior and household durable assets (e.g., number of rooms per capita, type of house ownership, house size per square meter, ownership of car, computer/laptop, freezer, dishwasher, TV, access to internet, etc.). The second part consisted of a validated Iranian version EuroQol 5-dimensions-3-level (EQ-5D-3L) questionnaire. It collects information on five dimensions of HRQoL *viz* . mobility, self-care, usual activity, pain/discomfort, and anxiety/depression, with three response levels for each dimension (no problem, some problem and extreme problem). The Iranian value set for EQ-5D-3L health states, extracted by visual analog scale (VAS), was used to calculate HRQoL of participants. More detailed information on this value set can be found elsewhere ^15^. The performance of this value set was compared against the UK VAS-based value set and the mean EQ-5D-3L scores were similar for the two value sets^16^.

We used poor-HRQoL as the outcome variable. We calculated the average HRQoL scores of participants. The participants scoring at least 0.1 points lower than the mean scores of the total sample were considered individuals with “poor-HRQoL”. This cut-off point is commonly used to detect moderate but clinically relevant differences ^17,18^. Participants with equal or more than the average HRQoL scores were considered individuals with “good-HRQoL”. To consider clinically moderate difference, we excluded 253 participants with the HRQoL scores between the average HRQoL scores and less than 0.1 below the average HRQoL scores. The dependent variable for the analysis was a binary variable of whether or not the participant had poor-HRQoL. Similar to previous studies ^7,8,17,19,20^, we included age, sex, marital status, SES, employment status, health insurance coverage, physical activity, smoking behavior, the presence of chronic health condition(s) as explanatory variables of poor-HRQoL in the decomposition analysis. We used detailed guidelines set out in the literature^21,22^ to construct a wealth index (WI), as a measure of SES, using a principal component analysis (PCA)^21,23^. We entered those assets and housing characteristics expected to be highly associated with households’ wealth (e.g., number of rooms per capita, type of house ownership, house size per square meter, ownership of car, TV, computer/laptop, freezer, dishwasher, microwave, vacuum cleaner, etc.) in the PCA. Based on the WI values, participants were divided into five SES groups (quintiles), from poorest to richest.

### Statistical analysis

We used the concentration index (C) approach^24^ to quantify socioeconomic-related inequalities in poor-HRQoL among the general population in Kermanshah, Iran. The C is based on the concentration curve. The concentration curve plots the cumulative percentage of participants ranked by SES (e.g., WI) in the x-axis and the cumulative percentage of a health variable of interest (poor-HRQoL) in the y-axis. The C is considered as twice the area between line indicating perfect equality and concentration curve. The values of the index vary between -1 and +1. The negative (positive) sign of the C indicates that the concentration curve lies above (under) the line of perfect equality and poor-HRQoL is more concentrated among the poor (wealthy). The zero value suggests, “perfect equality”^25^.

The following formula was used to calculate the C^26^:

(2) $$\text{C}=\frac{2\text{*cov}\left({\text{y}}_{\text{i}}{\text{ r}}_{\text{i}}\right)\;}{\text{μ}},$$

where µ indicates the mean of the health variable interest (i.e., poor-HRQoL) for the total sample, yi denotes the outcome variable for individual i, ri is the fractional rank in the SES distribution for the individual i. As the outcome variable in the study is binary, the minimum and maximum of the C are not between -1 and +1 and depend on µ. As per Wagstaff^27^, we normalized the C by multiplying by $\frac{1}{1-\mu }$ (i.e., $C_{n}=\frac{C}{1-\mu }$).

We decomposed the C to identify the contribution of each determinant (explanatory) variable to the wealth-related inequality in poor-HRQoL. If we have the following linear regression model linking our poor-HRQoL variable, y, to a set of k explanatory factors, xk :

(3) $$y=\alpha +\underset{k}{\sum }\beta_{k}\;x_{k}+\;\varepsilon .$$

The C for poor-HRQoL, y, can be decomposed as follows^28^:

(4) $$C=\underset{k}{\sum }\left(\frac{\beta_{k}{\bar{x}}_{k}}{\mu }\right)C_{k}+\frac{GC_{\varepsilon }}{\mu }$$

Where C shows the concentration index for health outcome (i.e., poor-HRQoL), ${\bar{x}}_{k}$ is the mean of explanatory variable xk, Ck is the C for xk, defined analogously to the C and $\frac{\beta_{k}{\bar{x}}_{k}}{\mu }$ is the elasticity of poor-HRQoL with respect to the explanatory variable xk. The $\underset{k}{\sum }\left(\frac{\beta_{k}{\bar{x}}_{k}}{\mu }\right)C_{k}$ indicates the contribution of explanatory factor xk to the C. A negative (positive) contribution of an explanatory factor to the C demonstrates that the wealth-related distribution of the factor and the association between the relevant factor and poor-HRQoL contributes to a lower likelihood of poor-HRQoL among the poor (rich). The last term, $\frac{GC_{\varepsilon }}{\mu }$, is the residuals component and reflects the socioeconomic-related inequality in health outcome that cannot be explained by systematic variation in xk across wealth groups^25^.

The normalized C can be decomposed using the following formula:

(5) $$C_{n}=\frac{C}{1-\mu }=\frac{\sum_{k}\left(\frac{\beta_{k}{\bar{x}}_{k}}{\mu }\right)C_{k}}{1-\mu }+\frac{\frac{GC_{\varepsilon }}{\mu }}{1-\mu }.\;\;$$

Since poor-HRQoL is a binary variable, we used marginal effects obtained from a logit model in the decomposition analysis ^25^. All analyses were performed in Stata ver. 14.2.

### Ethical statement

The verbal consent was obtained from each participant after explaining the purpose of the study. Each participant was also informed that s/he has the right to terminate the data collection process at any point. Those who did not provide consent to participate were excluded from the study. Data were collected anonymously and was only used for research. The Ethics Committee of the Deputy of Research at Kermanshah University of Medical Sciences (KUMS.REC.1396.458) approved the study.

## Results

Overall, 1730 adults aged 18-65 yr were included in the study, of which 1058 (61.2%) were men. The mean age ± (SD) of the respondents was 36.7 ± (12.6) yr. The majority of the study population was married (60.2%). Approximately, 18.4% of participants were current smokers and 14.3% of the study population had at least one chronic health condition. The average HRQoL scores ± (SD) was 0.65 ± (0.25) and the overall prevalence of poor-HRQoL was 35.3% (95% confidence interval [CI]; 33.1 to 37.6%) (Table 1).

**Table 1:** Descriptive statistics of variables included in the study (n =1730)

**Explanatory variables**	**Total**		**Poor-HRQoL** ^a^	
	**Number**	**Percent**	**Number**	**Percent**
Age groups (yr)				
18-30	894	51.7	195	21.8
31-45	396	22.9	142	35.7
46-65	440	25.4	274	62.3
Gender				
Male	1058	61.2	368	34.8
Female	672	38.8	243	36.2
Marital status				
Single	592	34.2	120	20.3
Married	1041	60.2	426	40.9
Divorce, separated and widows	97	5.6	65	67.0
Socioeconomic status (Household wealth)		
1st quintile (lowest)	346	20	196	56.6
2nd quintile	346	20	134	38.7
3rd quintile	346	20	111	32.1
4th quintile	347	20.1	85	24.5
5th quintile (highest)	345	19.9	85	24.6
Employment status				
Unemployed	664	38.4	247	37.2
Self-employed	619	35.8	205	33.1
Employed	370	21.4	104	28.1
Retired	77	4.4	55	77.4
Health insurance coverage				
Yes	1384	80	431	31.4
No	346	20	180	52.0
Smoking status				
Never	1330	76.9	398	29.9
Former	82	4.7	47	57.3
Current	318	18.4	166	52.2
Physical activity				
Inactive	254	14.6	176	69.3
Moderately active	527	30.5	258	49.0
Active	949	54.9	177	18.6
Chronic health condition(s)		
Yes	248	14.3	197	79.4
No	1482	85.7	414	27.9

^a^ Poor-HRQoL: Poor health-related quality of life

**Figure 1:**
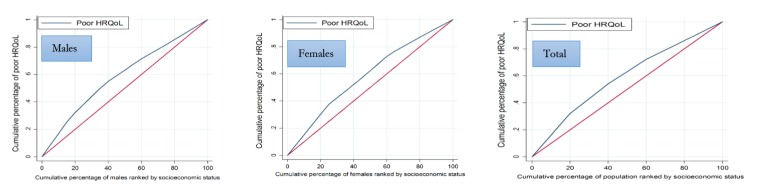
Concentration curve for poor-HRQoL among males, females and both sexes

The C_n_ for poor-HRQoL among males and females were -0.275 and -0.271, respectively. This figure for total samples was estimated to be -0.274. The poor-HRQoL is more concentrated among participants with lower SES (*P* <0.001). The concentration curves for poor-HRQoL lie above the line of perfect equality for males, females and total samples; suggesting that poor-HRQoL was more prevalent among the poor adults (Table 2 and Figure 1).

**Table 2:** Normalized concentration index in poor-HRQoL

**Sample**	**C**_n_ **(95% Confidence Interval)**
Male	-0.274 (-0.328, -0.219)
Female	-0.275 (-0.345, -0.205)
Total	-0.271 (-0.358, -0.184)

Table 3 reports the contribution of explanatory variables to the wealth-related inequality in poor-HRQoL in Kermanshah city, Iran. The table presents 1) 1) marginal effect of each explanatory variable, 2) the elasticity of each explanatory variable, 3) the Ck for each independent variable, and 4) absolute and percentage contribution of each explanatory variable to the observed wealth-related inequality in poor-HRQoL.

**Table 3:** Results of the decomposition analysis of inequality in poor-HRQoL

**Variables**	**Marginal effects**	**Mean (**xk)	**Elasticity**	**Concentration Index (C**_k_ **)**	**Absolute Contribution**	**Percentage Contribution**	**Summed Percentage Contribution**
Age groups (yr)							4.2
18-30	1.000	0.517	1.000	1.000	1.000	1.00	
31-45	0.116	0.229	0.074	-0.008	-0.000	0.20	
46-65	0.207	0.254	0.149	-0.073	-0.011	4.00	
Gender							-0.02
Male	1.000	0.612	1.000	1.000	1.000	1.00	
Female	-0.001	0.388	-0.001	-0.054	-0.000	-0.02	
Marital status							3.1
Single	1.000	0.342	1.000	1.000	1.000	1.00	
Married	0.070	0.602	0.123	-0.031	-0.004	1.37	
Divorce, separated and widows	0.125	0.056	0.020	-0.239	-0.005	1.73	
Socioeconomic status (Household wealth)							39.2
1st quintile (lowest)	0.190	0.200	0.108	-0.804	-0.086	31.6	
2nd quintile	0.113	0.200	0.064	-0.400	-0.026	9.30	
3rd quintile	0.083	0.200	0.047	0.000	-0.000	0.00	
4th quintile	0.021	0.201	0.012	0.401	0.005	-1.70	
5th quintile (highest)	1.000	0.199	1.000	1.000	1.000	1.00	
Employment status							2.7
Unemployed	1.000	0.383	1.000	1.000	1.000	1.00	
Self-employed	-0.096	0.358	-0.097	-0.062	0.006	-2.20	
Employed	-0.072	0.214	-0.044	0.384	-0.016	6.10	
Retired	0.159	0.045	0.020	0.162	0.003	-1.20	
Health insurance coverage							9.8
Yes	1.000	0.800	1.000	1.000	1.000	1.00	
No	0.202	0.200	0.114	-0.236	-0.027	9.8	
Smoking status							5.8
Never	1.000	0.769	1.000	1.000	1.000	1.00	
Former	0.229	0.047	0.030	0.135	0.004	-1.50	
Current	0.210	0.184	0.109	-0.184	-0.020	7.30	
Physical activity							14.6
Inactive	0.212	0.305	0.183	-0.066	-0.012	4.30	
Moderately active	0.355	0.147	0.148	-0.190	-0.028	10.3	
Active	1.000	0.548	1.000	1.000	1.000	1.00	
Chronic health condition(s)						12.9
Yes	0.364	0.143	0.147	-0.222	-0.033	11.9	
No	1.000	0.857	1.000	1.000	1.000	1.00	
Total explained					-0.250		91.1
Residual					-0.024		8.9
Total					-0.274		100

Older age was associated with higher probability of poor-HRQoL (the positive and significant sign of marginal effects for older adults). Compared with women, men had 0.1 percentage point higher probability of having poor-HRQoL. Lack of health insurance was associated with 20.2 percentage point higher probability of poor-HRQoL. Lower SES (household wealth) was associated with higher probability of poor-HRQoL among adults. Compared with never smokers, former and current smokers had 22.9% and 21% percentage point higher probability of poor-HRQoL.

Results of the C_k_ indicated that socio-demographic and socioeconomic characteristics, behavioral risk factors such as older age, women, married, self-employed, being uninsured, current smoker, inactive or moderately active and having chronic health condition(s) were more concentrated among the poor, whereas characteristics such as being employed, retired and former smokers were concentrated among the rich.

The largest contributor to the observed wealth-related inequality in poor-HRQoL was wealth index itself (39.2%). Besides wealth, physical activity, the presence of chronic health condition(s) and being uninsured were found to be main determinants of socioeconomic-related inequality in poor-HRQoL. The negative contributions of physical activity, the presence of chronic health condition(s) and being uninsured to wealth-related inequalities in poor-HRQoL indicated that the wealth-related distribution of these variables among adults in the sampled population and the association between these factors and poor-HRQoL led to the concentration of poor-HRQoL among less-wealthy adults. The results suggested 8.9% of wealth-related inequality in poor-HRQoL were not explained by the explanatory variables included in the model.

## Discussion

To date, some studies (e.g.,^8,14^) examined factors affecting HRQoL across different population groups in Iran. However, there is no study that quantifies and decomposes socioeconomic-related inequality in HRQoL in Iran. We measured and identified factors affecting wealth-related inequality in poor-HRQoL in the west of Iran.

The descriptive statistics results indicated that 35.3% of adults in Kermanshah city have poor-HRQoL. The findings from the concentration index and curve revealed that there were statistically significant pro-rich inequalities in poor-HRQoL among men, women and both sexes combined. Poor-HRQoL is more prevalent among the poor men and women. While the prevalence of poor-HRQoL among poorest SES group (lowest wealth quintile) was 56.6%, this figure was 24.6% for the richest group (highest wealth quintile). Studies in other countries also showed positive gradient between SES and poor-HRQoL. For example, there was an inverse association between SES and poor-HRQoL among adults aged 25 yr and older ^10^. Similarly, using a cross-sectional survey from Swedish population, the prevalence of poor-HRQoL among individuals with lower educational attainment (9 yr and under) was 43%, while the corresponding figure was 26% for higher educational attainment (more than 12 yr) individuals^17^. Lower HRQoL score was associated with lower SES in Ilam City in Iran^29^.

We found statistically significant positive associations between poor-HRQoL and older age, smoking behavior, physical inactivity, lack of health insurance, the presence of chronic health condition(s) and lower SES. These findings are consistent with the results reported by other studies. For example, smoking and physical inactivity led to the lower HRQoL among Iranian adults^8^. Another study observed negative impact of smoking on HRQoL in Iran ^30^. An inverse association between having a chronic health condition(s) and HRQoL is well-documented in the current literature^31-33^. Similar to our findings, having health insurance coverage was significantly associated with lower poor-HRQoL^7^. This association can be explained by the fact that individuals with health insurance coverage may have appropriate and timely access to healthcare services, which, in turn, may improve HRQoL of insured individuals.

The decomposition analysis of wealth-related inequality in poor-HRQoL revealed that wealth was the main contributor to the observed inequality. There are many reasons for the negative association between SES (as measured by household wealth) and poor-HRQoL. Wealth inequality can be translated into inequality in health through inequalities in resources and material opportunities such as nutrition, housing, and healthcare utilization ^34-36^. Income-related inequality in health was measured and decomposed in Shiraz, Iran^12^. Similar to our study, the latter study reported income (SES) as the most important factor contributing to pro-rich inequality in health (39.92% for general health and 39.82% for mental health). Lower SES (income, occupational status, and education level) is significantly related to the lower HRQoL in Germany ^37^. Moreover, individuals with lower SES had lower HRQoL^38^. Differences in the financial barriers to access healthcare services across different socioeconomic groups might have contributed to the observed association.

The decomposition analysis recommended that, besides SES, physical inactivity, the presence of chronic health condition(s) and absence of health insurance were the main drivers of the concentration of poor-HRQoL among the poor in Kermanshah, Iran. Using a sample of 5537 adults, aged between 40 to 60 yr, low level of physical activity was related to poor-HRQoL in England ^39^. Similarly, having a higher level of physical activity was negatively associated with having poor-HRQoL for men and women and for both sexes combined ^40^.

Our study was subject to several limitations and the results should be interpreted with caution. Firstly, the study used convenience sampling method to select study participants in Kermanshah city; thus, the generalizability of the study findings is limited. Secondly, this study was cross-sectional and therefore we were unable to establish causal relationships between the explanatory variables and poor-HRQoL. Third, information on HRQoL and its determinants were self-reported and subject to recall bias.

## Conclusions

Our study showed a pro-rich inequality in poor-HRQoL in Kermanshah, Iran. Wealth, physical inactivity, the presence of chronic health condition(s) and lack of health insurance were the four main factors contributing to inequality in poor-HRQoL. Therefore, effectiveness programs that aim to address social determinants of health and increase physical activity, reduce smoking prevalence, increase health insurance coverage for the poor may reduce socioeconomic-related inequality in poor-HRQoL in Iran.

## Acknowledgements

The authors gratefully acknowledge the Research Council of Kermanshah University of Medical Sciences.

## Conflict of interest statement

The authors have no conflicts of interest to declare for this study.

## Funding

The study was funded and supported by the Research Deputy of Kermanshah University of Medical Sciences (Grant Number: 96491).

## Highlights

- The prevalence of poor-HRQoL among men and women was 34.8 and 36.2, respectively.
- The poor-HRQoL is more concentrated among lower SES adults.
- The prevalence of poor-HRQoL among the poorest quintile groups was 56.6%.
- The prevalence of poor-HRQoL among the wealthiest quintile group was 24.6%.

## References

[1] Marmot M (2005). Social determinants of health inequalities. Lancet.

[2] Goldman N (2001). Social inequalities in health. Ann N Y Acad Sci.

[3] Marmot M (2005). Social determinants of health inequalities. Lancet.

[4] Marmot M, Ryff CD, Bumpass LL, Shipley M, Marks NF (1997). Social inequalities in health: next questions and converging evidence. Soc Sci Med.

[5] Hajizadeh M, Mitnitski A, Rockwood K (2016). Socioeconomic gradient in health in Canada: Is the gap widening or narrowing?. Health Policy.

[6] Hosseinpoor AR, Williams JAS, Itani L, Chatterji S (2012). Socioeconomic inequality in domains of health: results from the World Health Surveys. BMC Public Health.

[7] Kazemi Karyani A, Rashidian A, Emamgholipour Sefiddashti S, Akbari Sari A (2016). Self-reported health-related quality of life (HRQoL) and factors affecting HRQoL among individuals with health insurance in Iran. Epidemiol Health.

[8] Rezaei S, Hajizadeh M, Kazemi A, Khosravipour M, Khosravi F, Rezaeian S (2017). Determinants of health-related quality of life in Iranian adults: evidence from a cross-sectional study. Epidemiol Health.

[9] Mielck A, Reitmeir P, Vogelmann M, Leidl R (2012). Impact of educational level on health-related quality of life (HRQL): results from Germany based on the EuroQol 5D (EQ-5D). Eur J Pub Health.

[10] Matute I, Burgos S, Alfaro T (2017). Socioeconomic Status and Perceived Health-related Quality of Life in Chile. Med Rev.

[11] Burström K, Johannesson M, Diderichsen F (2001). Health-related quality of life by disease and socio-economic group in the general population in Sweden. Health Policy.

[12] Ramezani Doroh V, Vahedi S, Arefnezhad M, Kavosi Z, Mohammadbeigi A (2015). Decomposition of Health Inequality Determinants in Shiraz, South-west Iran. J Res Health Sci.

[13] Burström K, Johannesson M, Diderichsen F (2001). Swedish population health-related quality of life results using the EQ-5D. Qual Life Res.

[14] Ghafari R, Rafiei M, Taheri Nejad M (2014). Assessment of health related quality of life by SF-36 version 2 in general population of Qom city. Arak Med Univ J.

[15] Goudarzi R, Zeraati H, Sari AA, Rashidian A, Mohammad K (2016). Population-based preference weights for the EQ-5D health states using the visual analogue scale (VAS) in Iran. Iran Red Crescent Med J.

[16] Kiadaliri AA (2017). A comparison of Iran and UK EQ-5D-3L value sets based on visual analogue scale. Int J Health Policy Manag.

[17] Djärv T, Wikman A, Johar A, Lagergren P (2013). Poor health-related quality of life in the Swedish general population: The association with disease and lifestyle factors. Scand J Public Health.

[18] Osoba D, Rodrigues G, Myles J, Zee B, Pater J (1998). Interpreting the significance of changes in health-related quality-of-life scores. J Clin Oncol.

[19] Taira DA, Seto TB, Ho KK, Krumholz HM, Cutlip DE, Berezin R (2000). Impact of smoking on health-related quality of life after percutaneous coronary revascularization. Circulation.

[20] Mulder I, Tijhuis M, Smit HA, Kromhout D (2001). Smoking cessation and quality of life: the effect of amount of smoking and time since quitting. Prev Med.

[21] Vyas S, Kumaranayake L (2006). Constructing socio-economic status indices: how to use principal components analysis. Health Policy Plan.

[22] Kolenikov S, Angeles G (2009). Socioeconomic status measurement with discrete proxy variables: Is principal component analysis a reliable answer?. Rev Income Wealth.

[23] Houweling TA, Kunst AE, Mackenbach JP (2003). Measuring health inequality among children in developing countries: does the choice of the indicator of economic status matter ?. Int J Equity Health.

[24] Wagstaff A, Paci P, Van Doorslaer E (1991). On the measurement of inequalities in health. Soc Sci Med.

[25] O’donnell O, Van Doorslaer E, Wagstaff A, Lindelow M. Analyzing health equity using household survey data. Washington DC: World Bank. 2008.

[26] Kakwani Nanak C, Kakwani Nanak C. Income inequality and poverty: methods of estimation and policy applications: Oxford University Press; 1980.

[27] Wagstaff A (2005). The bounds of the concentration index when the variable of interest is binary, with an application to immunization inequality. Health Econ.

[28] Wagstaff A, Van Doorslaer E, Watanabe N (2003). On decomposing the causes of health sector inequalities with an application to malnutrition inequalities in Vietnam. J Econom.

[29] Menati W, Baghbanian A, Asadi-Lari M, Moazen J, Menati R, Sohrabivafa M (2017). Health-Related Quality of Life and Socioeconomic Status: Inequalities among Adults in West of Iran. Iran Red Crescent Med J.

[30] Rezaei S, Karami Matin B, Kazemi Karyani A, Woldemichael A, Khosravi F, Khosravipour M (2017). Impact of smoking on health-related quality of life: A general population survey in West Iran. Asian Pac J Cancer Prev.

[31] Augustussen M, Sjøgren P, Timm H, Hounsgaard L, Pedersen ML (2017). Symptoms and health-related quality of life in patients with advanced cancer-A population-based study in Greenland. Eur J Oncol Nurs.

[32] Hajian-Tilaki K, Heidari B, Hajian-Tilaki A (2016). Solitary and combined negative influences of diabetes, obesity and hypertension on health-related quality of life of elderly individuals: A population-based cross-sectional study. Diabetes Metab Syndr.

[33] Kitaoka M, Mitoma J, Asakura H, Anyenda OE, Nguyen TTT, Hamagishi T (2016). The relationship between hypertension and health-related quality of life: adjusted by chronic pain, chronic diseases, and life habits in the general middle-aged population in Japan. Environ Health Prev Med.

[34] Esmailnasab N, Hassanzadeh J, Rezaeian S, Barkhordari M (2014). Use of health care services and associated factors among women. Iran J Public Health.

[35] Hajizadeh M, Connelly LB, Butler JR, Khosravi A (2012). Unmet need and met unneed in health care utilisation in Iran. Int J Soc Econ.

[36] Abbott S (2002). Prescribing welfare benefits advice in primary care: is it a health intervention, and if so, what sort?. J Public Health.

[37] Klein J, Hofreuter-Gätgens K, Lüdecke D, Fisch M, Graefen M, von dem Knesebeck O (2016). Socioeconomic status and health-related quality of life among patients with prostate cancer 6 months after radical prostatectomy: a longitudinal analysis. BMJ Open.

[38] Huguet N, Kaplan MS, Feeny D (2008). Socioeconomic status and health-related quality of life among elderly people: results from the Joint Canada/United States Survey of Health. Soc Sci Med.

[39] Anokye NK, Trueman P, Green C, Pavey TG, Taylor RS (2012). Physical activity and health related quality of life. BMC Public Health.

[40] Buder I, Zick C, Waitzman N (2016). Health‐Related Quality of Life Associated With Physical Activity: New Estimates by Gender and Race and Ethnicity. World Med Health Policy.

